# Adolescent triple-negative breast cancer with germline pathogenic variants in both *BRCA1* and *TP53 genes*: A case report

**DOI:** 10.3389/fonc.2022.970641

**Published:** 2022-11-28

**Authors:** Dongmei Chen, Chenyang Zhang, Mengqi Yuan, Ying Zhang, Qing Liu, Donggui Wan

**Affiliations:** ^1^ Department of Integrative Oncology, China-Japan Friendship Hospital, Beijing, China; ^2^ Graduate School, Beijing University of Chinese Medicine, Beijing, China

**Keywords:** triple-negative breast cancer, TP53, BRCA1/2, young breast cancer, gene mutation

## Abstract

Almost 5-10% of breast cancer results from inherited genetic pathogenic variants. Patients with pathogenic variants in high-penetrance genes such as *TP53*, *BRCA1* and *BRCA2* are susceptible to breast cancer. Moreover, nearly 80% of *BRCA* pathogenic variants carriers are diagnosed with breast cancer at a young age before menopause. There is currently no report of early onset breast cancer with germline pathogenic variants in both *BRCA1* and *TP53* genes. Here, we report a case of a 14-years-old female diagnosed with triple-negative breast cancer with a family history of malignant tumors. The cancer metastasized to multiple lymph nodes 1 year and 4 months after surgery, and the progression-free survival after subsequent chemotherapy and surgery has been 2 years and 10 months. The patient’s white blood cells were screened against a panel of 11 cancer-related genes, and both germline pathogenic variants in *BRCA1* and *TP53* were identified. Genetic tests of her family members revealed the same pathogenic variants in *BRCA1* in her father and brother, but *BRCA1* pathogenic variants wasn’t shown in other family members. The case indicates that genetic testing needs be performed in early onset breast cancer to confirm inherited risk, and if a germline pathogenic variant is identified, tailored therapeutic interventions and preventive interventions should be taken and genetic testing is recommended for relatives.

## Introduction

The incidence of breast cancer ranks first among all cancers according to the report released by International Association of Cancer Research (1). The median age is 61 years old at which breast cancer was diagnosed, and only 7% of the cases occur in young women (2). The hormone receptor positive(HR+)/human epithelial growth factor receptor 2 positive(HER2+), triple-negative and HR-/HER2+ subtypes are more prevalent among the younger breast cancer patients, who are also more likely to present with more advanced stages of the disease (III/IV) compared to older women (3). In addition, young age is an independent risk factor for disease recurrence (4). Breast cancer is relatively rare among adolescents, defined as ages between 10-19 years according to WHO, and very few reports exist for adolescent breast cancer (3, 5, 6). Early onset breast cancers are often familial, and about 50% of the patients under the age of 30 carry germline pathogenic variants(PVs) in *BRCA1*, *BRCA2* or *TP53* (7, 8). The most common familial cancer syndromes are the hereditary breast and ovarian cancer syndrome (HBOC), which is associated with pathogenic variants in *BRCA1* or *BRCA2*, and Li-Fraumeni syndrome(LFS), which is associated with germline PV in *TP53* (9). HBOC is characterized by multiple family members with breast cancer, ovarian cancer, or both. While, LFS, which was first reported in 1969 (10), is characterized by increased susceptibility to premenopausal breast cancer, soft tissue sarcomas, brain tumors, leukemias and adrenocortical carcinomas (11). Therefore, it’s important for oncology practitioners to take a detailed family history during the initial visit. Be aware of hereditary patterns or key features of inherited predisposition for instance early age at onset triggers the evaluation for familial cancer syndromes. Here, we report an early onset triple-negative breast cancer(TNBC) with germline PVs in both *BRCA1* and *TP53* genes.

## Case

Our patient is a 19-year-old girl, who was diagnosed with TNBC at the age of 14. She presented for consultation after metastasis and recurrence. The patient had no previous medical problems, but had a family history of cancer. Her paternal grandmother died of lung cancer at the age of 29 and her maternal grandfather died of nasopharyngeal carcinoma. Menarche had occurred at 9 years of age. Ultrasound imaging revealed a mass in the right breast when the patient was 14 years old. Then, she underwent protective and radical operation of the breast mass and sentinel lymph node biopsy in December 2017. The breast mass measured 3.2×1.8 cm, and histopathological examination revealed grade III invasive carcinoma of non-specific type, with ki-67 proliferation index of 70%, and estrogen receptor (ER), progesterone receptor (PR) and Her2/neu negative subtype. Eight sentinel nodes were retrieved using the blue dye and radioisotope methods, and no metastasis was detected. The patient was diagnosed with stage IIA invasive carcinoma of the right breast. She received 6 cycles of adjuvant chemotherapy with TEC (docetaxel 75mg/m^2^, epirubicin 75mg/m^2^ and cyclophosphamide 500mg/m^2^ on day 1, cycled every 21 days) regimen, followed by 25 sessions of adjuvant radiotherapy and 6 cycles of electron therapy. The adjuvant treatment was completed by the April of 2018.

**Figure 1:**
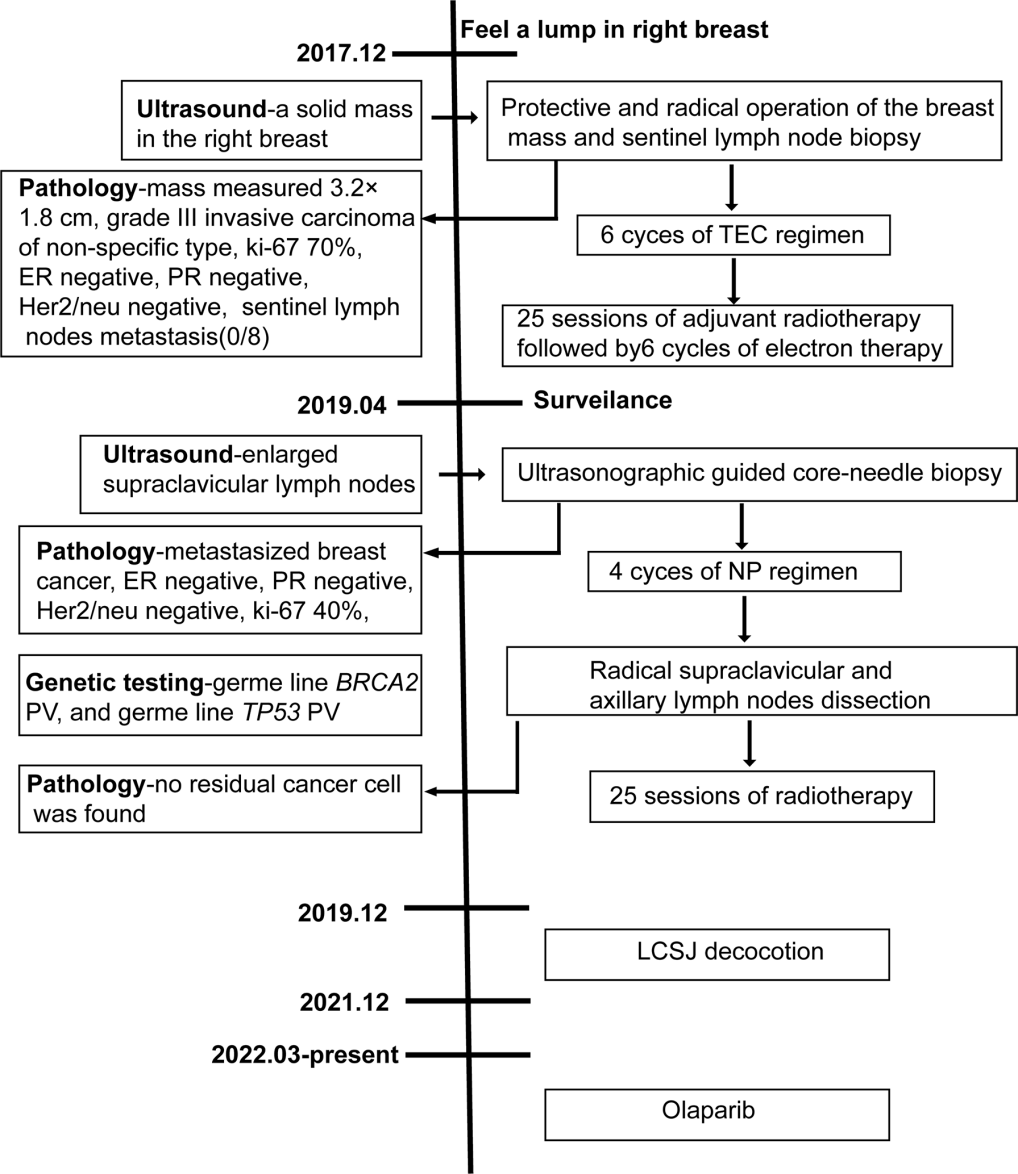
Case report timeline.

The disease-free survival (DFS) was 1 year and 4 months. Then, ultrasound examination during follow-up in April, 2019 indicated enlarged supraclavicular lymph nodes. Tissue sampling with core-needle biopsy under ultrasonographic guidance was performed later. Histologic evaluation of the tissue indicated metastasized breast cancer. Immunohistochemical staining revealed that the tumor cells were negative for ER, PR and Her-2, with ki-67 proliferation index of 40%. No visceral or brain metastasis was identified by computerized tomography (CT) scanning and brain magnetic resonance imaging (MRI). The patient received 4 cycles of NP(vinorelbine 25mg/m^2^ on day 1 and 8, cisplatin 40mg/m^2^ on day 1 and 8, cycled every 21 days) as first-line chemotherapy. No obvious side effect was observed during chemotherapy. Radical supraclavicular and axillary lymph nodes dissection was subsequently performed. No residual cancer cell was found in the resected tissue by histopathological examination, suggesting a pathological complete response(pCR). Then, 2 cycles of NP regimen followed by radiotherapy were performed after surgery. Right after conventional anti-cancer therapy, the patient started to take LCSJ (composed of *Mongolian snakegourd pericarp*, *edible tulip, catclaw buttercup root*, *safflower*, *rugose rose flower*, *shorthorned epimedium herb*, *radix astragali, tuckahoe, oyster shell, radix glycyrrhizae and cimicifugae rhizome* at a ratio of 10:5:10:3:5:5:5:5:10:3:2) decoction, a Chinese herbal formula used for anti-recurrence in TNBC. LCSJ treatment ended by December, 2021. Blood routine and liver and kidney function were monitored every 3 months, no adverse effect occurred. Then, she started to take Olaparib 300mg twice a day since March, 2022. The patient remained disease free at the end of the follow up(June 25, 2022). The PFS is 2 years and 10 months. A case report timeline was shown in **Figure 1**.

Given the early onset of breast cancer and a family history of cancer, genetic testing was conducted after metastasis. White blood cells were collected from the patient (proband, III2) and subjected to a next generation sequencing (NGS)-based genetic test using a panel of 11 cancer-related genes. Variants were called by the third-party software GATK v3.7 (https://software.broadinstitute.org/gatk/) and SAM tools (http://github.com/samtools/samtools) were employed for identification of single nucleotide variations (SNVs) and small insertions and deletions (12). Variant annotation and interpretation were conducted by ANNOVAR (13). Annotation databases mainly included i) human population databases, such as the gnomAD (http://gnomad.broadinstitute.org/), the 1000 Genome Project (http://browser.1000genomes.org), the dbSNP (http://www.ncbi.nlm.nih.gov/snp), etc.; ii) in silico prediction algorithms, such as SIFT (http://sift.jcvi.org), FATHMM (http://fathmm.biocompute.org.uk), Mutation Assessor (http://mutationassessor.org), CADD (http://cadd.gs.washington.edu), SPIDEX (14), etc.; and iii) disease and phenotype databases, such as OMIM (http://www.omim.org), ClinVar (http://www.ncbi.nlm.nih.gov/clinvar), HGMD (http://www.hgmd.org), HPO (https://hpo.jax.org/app/), etc. Pathogenic variants were identified in *TP53* (chr17: 7673802C>T) and *BRCA1* (chr17: 43124030C>CT) (**Supplementary Table 1**).

**Figure 2:**
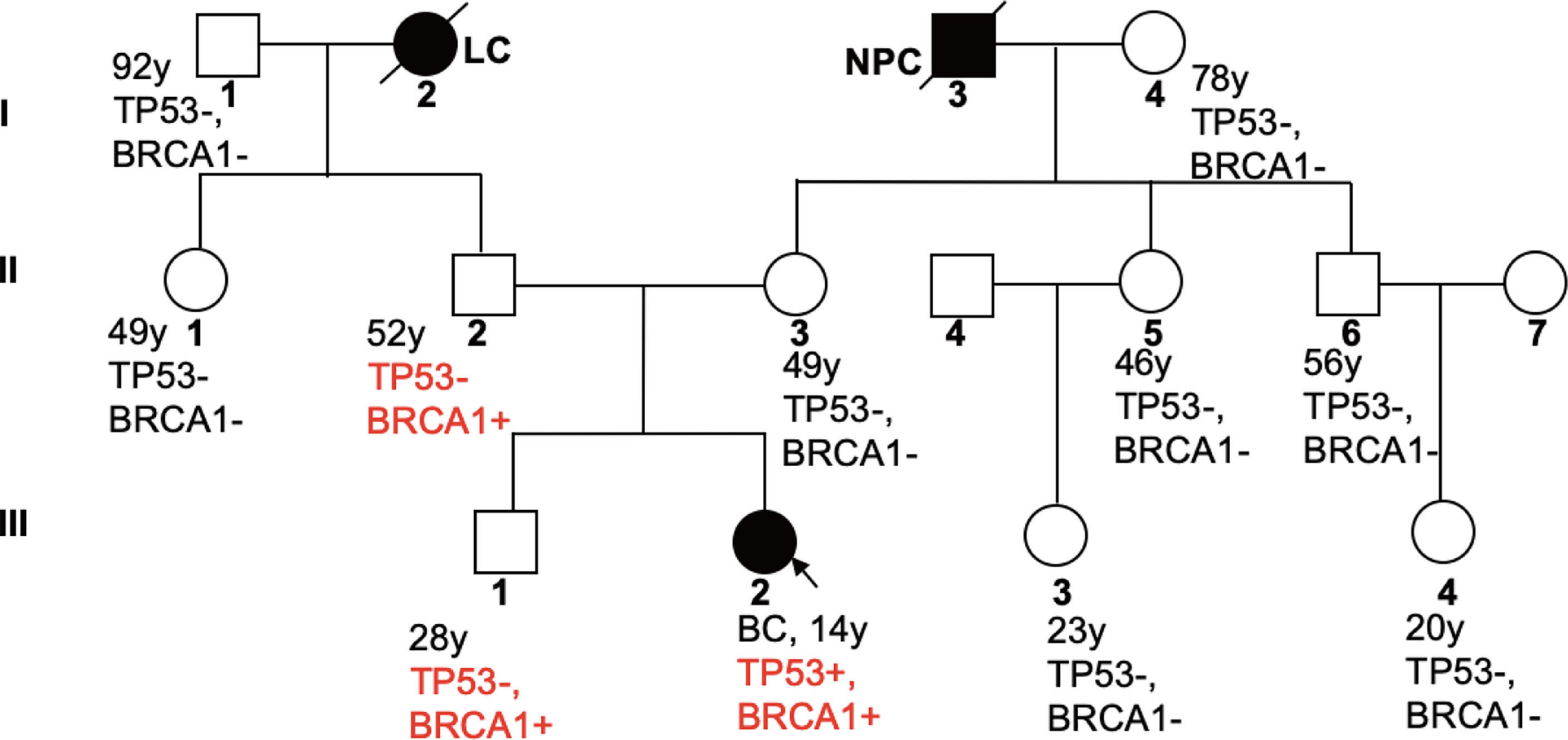
Familial Pedigree of case. The proband is indicated by an arrowhead. Squares represent males, circles represent females. Solid symbols represent affected individuals. Symbols with slash indicate deceased individuals. Age at cancer diagnosis is reported following the corresponding disease. LC, lung cancer; NPC, nasopharyngeal carcinoma.

In order to delineate the inheritance pattern of variants in her family, we also tested genes of the patient’s family members and identified the same PV in BRCA1 in the patient’s father and brother (II2, III1), but not in other family members. The family tree is shown in **Figure 2**.

## Discussion

This is the first case report of adolescent breast cancer with both *TP53* and *BRCA1* germline PVs. Jay et al. reported a case of adolescent breast cancer with *TP53* PV, who was also diagnosed with Li-Fraumeni syndrome in 2017 (12). Our patient has a family history of cancer, with her paternal grandmother dying of lung cancer at the age of 29 and her maternal grandfather from nasopharyngeal carcinoma. Therefore, we characterized the inheritance of both PVs by sequencing the sliva samples of family members. The 11-gene panel identified a PV with repeated insertion of adenine at base 66 of NM_007294.3 transcript of *BRCA1* gene that resulted in a frameshift variant of glutamic acid at position 23 (p. Glu23fs), and a pathogenic substitution variant (G>A) at nucleotide position 818 of *TP53* gene that caused replacement of arginine by histidine at position 273. The patient’s father and brother are carriers of the same *BRCA1* variant, indicating a paternal origin. While, *TP53* germline variant was only detected in the patient, which makes her the proband.

Breast cancer susceptibility genes including *TP53*, *BRCA*, *CDH1*,*PTEN*, *PALB2*, *ATM*, *CHEK2*, and so on. Among them, *TP53*, *BRCA*, *CDH1* and *PTEN* are defined as high-penetrance genes, while *PALB2*, *ATM* and *CHEK2* are usually regarded as moderate-penetrance genes. Certain genes are associated with familial cancer syndromes, such as *BRCA* genes related HOBC and *TP53* related LFS. However, the prevalence of HOBC or LFS in China hasn’t yet been reported. *BRCA2* germline PVs ranks first (3.5%) among all germline PVs reported by an investigation conducted in 8085 unselected breast cancer patients in China (15). *The* PVs rate is 1.5% *in BRCA1* and 3.5% in *BRCA2*. However, the variant rate of *BRCA1* and *BRCA2* is as high as 24.1% in patients with a family history of breast cancer (15). Moreover, 16% of TNBC in this study were reported to have PVs in either *BRCA1* or *BRCA2.* Another study determined *TP53* germline variants in a large cohort of 10053 breast cancer in Chinese populations. The results revealed that the prevalence of PVs in *TP53* is 0.5%. Yet, it increased to 3.8% in very early onset breast cancer (≤30 years old) (16). In this case, the patient had a family history of cancer and was diagnosed as TNBC at the age of 14. As such, genetic testing is essential for breast cancer patients to identify inherited PVs in cancer susceptibility genes, which is not only a diagnostic step to confirm inherited risk, but also can affect management of the patient and enable cascade genetic evaluation of family members.

Compared with sporadic breast cancer, patients with germline *BRCA* PVs are more likely to develop contralateral breast cancer, the cumulative risk 20 years after breast cancer diagnosis was 40% for *BRCA1* and 26% for *BRCA2* (17). Population-based studies also revealed that lifetime risk of developing ovarian cancer also significantly elevated for *BRCA1/2* carriers, (18), about 44% for *BRCA1* and 17% for *BRCA2* (17). In addition to epidemiological factors, the histopathological characters are also associated with *BRCA* PVs. Breast cancers carry *BRCA1* PVs tend to be poorly differentiated, high-grade invasive ductal carcinomas, and they are more likely to be triple-negative. However, the frequency of ER and PR positivity in *BRCA2* PVs is similar to sporadic breast cancer (18–20). Thus, management of breast cancer with *BRCA* germline PVs differs from sporadic breast cancer. Usually whether to perform adjuvant radiotherapy and the dosage of radiotherapy need to be considered as *BRCA* PVs carriers may be at elevated risk for radiation associated toxicity, moreover, being at higher risk for contralateral breast cancer due to radiation scatter (21). Though mastectomy and contralateral risk-reducing mastectomy are recommended for *BRCA* PV carriers, considering the extremely young age of breast cancer onset, psychological factors, and the willing of patient, our patient underwent conserved breast therapy and radiotherapy subsequently. Additionally, how to appropriately address the risk of ovarian cancer and fertility preservation arise as questions for this *BRCA2* gene PV carrier. Ovarian function suppression was administrated during adjuvant treatment. *BRCA1* or *BRCA2* mutation also affects the treatment regimens for breast cancer. As *BRCA* gens are tumor suppressor genes that mediate DNA double-strand break repair through a homologous recombination mechanism (13). Poly(adenosine diphosphate-riose) polymerase inhibitors(PARPi) exploit the principle of synthetic lethality to selectively kill cancer cells that have deficiency in homologous recombination repair (22). PARPi such as olaparib and talazoparib have been proved to be beneficial for breast cancer carry BRCA1 or BRCA2 mutation in neoadjuvant, adjuvant as well as salvage therapy in later stage (23–26). Our patient wasn’t able to take PARPi after adjuvant therapy or right after recurrence as salvage therapy till March, 2022 for financial reason.

Apart from BRCA, germline PV in *TP53* was identified in this case as well. *TP53* regulates the cellular response to diverse stresses and maintains genomic integrity, which is critical to its role in tumor suppression (27). The loss of BRCA1/2 function leads to disruption of the homologous recombination based DNA repair and, as a result, to the large-scale genomic instability (28). Genomic instability induced by BRCA PV might facilitate mutation of the remaining wild-type TP53 allele in BRCA1-deficient cells. Alternatively, because BRCA1 genes are also involved in the G2-M and spindle assembly checkpoints, loss of heterozygosity at the *TP53* locus might occur more efficiently in *BRCA1*-deficient cells (29).The variant of *TP53* increase the lifetime risk of developing cancer to 75% in males and almost 100% in females (30). *TP53* is also an indicator of cisplatin sensitivity (16). Thus, cisplatin-related regimens are recommended during disease progression. This patient achieved pCR with 4 cycles of NP regimen after lymph nodes metastasis, indicating being sensitive to cisplatin-based regimens. First line PFS reached 2 years and 10 months after conventional salvage treatment.

Since *BRCA* and *TP53* germline PV are involved in hereditary cancer. Thus, extensive cancer surveillance, for instance whole body MRI (rapid non-contrast examinations), is recommended for this patient to monitor tumor recurrence and metastasis, as well as any possibility of ovarian cancer and pancreatic cancer.

Meanwhile, genetic testing is recommended for family members to assess possible inherited cancer risk, and if possible genetic counselling is recommended to interpret genetic testing result and to provide options for management.

In conclusion, we have reported a case of an adolescent TNBC with germline PVs in *BCRA1* and *TP53* with a well-characterized inheritance pattern. She was diagnosed at the age of 14, and tumor cells metastasized to lymph nodes 1 year and 4 months after surgery. The first-line PFS is currently 2 years and 10 months, which portends favorable prognosis. The case underscores the importance of genetic risk evaluation in patient with a family history of cancer. And also, tailored therapeutic interventions and preventive interventions need to be administrated.

## Data availability statement

The raw data supporting the conclusions of this article will be made available by the authors, without undue reservation.

## Ethics statement

The studies involving human participants were reviewed and approved by Medical Ethics Committee of China-Japan Friendship Hospital. The patients/participants provided their written informed consent to participate in this study. Written informed consent was obtained from the individual(s) for the publication of any potentially identifiable images or data included in this article.

## Author contributions

DC and CZ were major contributor in writing the manuscript; MY and YZ contributed to the medical history taking; CZ and QL helped revised the manuscript. DW and DC contributed to the design of the work. All authors participated in the treatment of the patient. All authors read and approved the final manuscript.

## Funding

This work was supported by Natural Science Foundation of Beijing Municipality (7214295), Foundation for Young Scientist of China-Japan Friendship Hospital(2019-2-QN-63), personnel training program of China-Japan Friendship Hospital elite project (ZRJY2021-TD05), Beijing developmental funding of Traditional Chinese Medicine (JJ-2020-88) and Foundation of Wujieping(320.6750.2020-07-24).

## Acknowledgments

We thank Genetron Holdings Limited for providing the genetic testing for the family members of the patient.

## Conflict of interest

The authors declare that the research was conducted in the absence of any commercial or financial relationships that could be construed as a potential conflict of interest.

## Publisher’s note

All claims expressed in this article are solely those of the authors and do not necessarily represent those of their affiliated organizations, or those of the publisher, the editors and the reviewers. Any product that may be evaluated in this article, or claim that may be made by its manufacturer, is not guaranteed or endorsed by the publisher.
